# World’s Columbian Dental College—1893

**Published:** 1894-01

**Authors:** J. M. Walker


### THE DENTAL REGISTER.

VOL. XLVIII.] JANUARY, 1894. [No. 1

### SOCIETY PROCEEDINGS.

### World's Columbian Dental Congress—1893.

SCIENTIFIC WORK BY SECTIONS.

Reported for the DENTAL REGISTER by Mrs. J. M. Walker.

FRIDAY, AUGUST 18TH.—FIFTH DAY.—GENERAL SESSION.

(*Continued from page* 612, VOL. XLVII.)

The meeting was called to order by Dr. L. D. Shepard, at 12 M.

Without any preliminary proceedings, the President called for the first paper, entitled: "English Tube Teeth," by John Girdwood, L.D.S., Edinburgh; D.D.S., University of Pennsylvania; Edinburgh, Scotland.

In the absence of the essayist, the paper was read by Dr. A. O. Hunt. The essayist said: The absence of a description of tube teeth in any American work was cause for much surprise to European dentists, as they consider them superior to flat teeth in many cases. He described their several points of advantage, as follows:

1st. That they are much stronger than flat teeth, especially for service as masticators, being supported under the whole lower surface.

2d. In easy removal for repairs.

3d. In requiring no backing, thus eliminating the danger of warping the piece.

4th. In being of the same texture throughout, so that they can be ground down wherever this is required, and polished perfectly.

5th. Their cheapness, owing to their ease of adaptability.

6th. They are a faithful reproduction of the forms of the natural teeth, and, consequently, comfortable to the tongue, and are less bulky.

7th. They are more easily kept clean, because backings are done away with, and better supports substituted, which being surrounded by porcelain are out of the reach of any impurity.

For crown and bridge-work they have all the advantages already enumerated, and, in addition, they can be more perfectly and directly fitted to the root than any other form of porcelain crown. They retain when mounted for wear in the mouth, their translucency and natural appearance, so often destroyed by the gold backing.

The essayist described at length the tools required, and the process of mounting these teeth, as single crowns, with or without caps or bands for bridge-work, fixed or removable, etc. In the use of these teeth the pins are cemented in the teeth with molten sulphur, broken teeth being readily removed by the application of a moderate degree of heat. In conclusion, the essayist asked, "What more can an operator wish, when he has presented to him for use a tooth differing essentially from the natural one in but one respect—that it does not live? What more does the patient demand than that having his dental apparatus restored as far as utility is concerned, he is at liberty to laugh and chat to his heart's content, without betraying the fact that he owes his perfect comfort to art? It is no exaggeration to say that in the use of the tube tooth such a happy state of things is realized."

This paper was referred to Section 7 for discussion.

After the reading of this paper Dr. Shepard called Dr. Lyon to the chair. Dr. Lyon introduced Dr. Marshall, the Treasurer of the Congress, who stated that the number of foreign guests registered is 115; Americans, 982; making a total of 1097. The amount contributed so far is $13,231.95.

The Chairman then introduced Dr. Bryan, of Basle, Switzerland.

Dr. Bryan: I am glad of the opportunity to thank the dentists of Chicago and the administration of this Congress, as well as the committees, and those who have so generously provided for our entertainment here and so hospitably received us. I think all the foreign members will join me in thanking you, and expressing our gratitude for the success of the Congress.

Dr. Thos. Fillebrown, Boston, next read a paper, entitled "Hypnotic Suggestion as a Dental Obtundent and Sedative."

Dr. Fillebrown referred to the contrast between the action of the British Medical Association six years ago, when a paper on this subject was refused a hearing, and a statement in the *British Medical Journal* a year ago, that "Hypnotism has come to stay; it is deserving the attention of every thoughtful scientific man." Last year a paper presented before the American Medical Association on this subject was ruled out, while to-day the subject is chosen by the World's Columbian Dental Congress as the one most likely to interest the largest number of persons, the very large attendance justifying that choice.

The paper was largely a record of cases of the successful application of the principles of hypnotic suggestion, more especially as an obtundent for painfully sensitive dentine, the patient being fully conscious of all that was going on, and fully realizing the obtunding effect of the suggestion; also, in relieving the dread and terror of dental operations, so often a great hindrance to successful operation—its sedative influence on the nervous system, the patient resting during the operation, instead of being exhausted. The essayist recommends the careful study of "Bernheim's Suggestive Therapeutics" to all who are interested in the subject.

The paper was passed without discussion, owing to the lateness of the hour, and referred to Section 1.

After several announcements by the Secretary-General, the Congress adjourned to meet Saturday, at 11 A. M., for the final session.

### WORK OF THE SECTIONS, FRIDAY, AUGUST 18TH.

### SECTION 1.—ANATOMY AND HISTOLOGY.

The meeting was called to order by Dr. C. P. Robinson, of Mobile.

Discussion on Dr. Fillebrown's paper, entitled, "Hypnotic Suggestion as a Dental Obtundent and Sedative."

Several cases were related in which without any thought of hypnotism the results had been similar to those cited by Dr. Fillebrown, who, in substance, replied: I am indebted to the gentlemen for speaking of these cases. When you come to look back over your experience, three-quarters of you will see that you have practically been hypnotizers over a long series of years. You will find that you are in just the condition that a gentleman was who spoke to me a little while ago. He said: "Well, I have been doing just that thing right along, and did not know it." You have ignorantly practiced it, and I come here and declare to you a way by which you can use the method in a great many directions in which you have not used it before. You can all do this when you get home. You can experiment on the first patient you have, whether he knows anything about hypnotism or not. Two friends of mine in Boston have had a great deal of success in this way. Say to your patient: "If you will do as I say, you will find the pain very much relieved. You feel like resting." While you are saying that, if you will watch carefully the muscular tension, you will notice how it relaxes in less time than I have been rehearsing it to you. Then go on and do just as I tell you. Do it quietly, and you may be sure that it will come about and help you wonderfully.

Dr. McKellops: How many men have that power? I have paid a great deal of attention to this. It was illustrated at the Mississippi Valley Dental Association ten years ago. It is all very well for certain people to do that, but I can take any patient, and by kindness and gentleness in my operation, do anything that Dr. Fillebrown does, without any hypnotism. If you treat your patients kindly, and operate gently, you will get the same results as my friend gets from hypnotism. Many men in the profession lack that kindness and gentleness in operations that would make them successful. I used to use ether and chloroform to a great extent. I gave it to man, woman, and child, and I am surprised that I did not kill some of them. I gave it promiscuously, but I have quit doing so. I have done away with those things. I can, by careful operation and handling my patients with care, accomplish anything that can be done with anæsthetics. I want my patients to know what I am doing. I have tried it until I have worn myself out, and I have seen all there is to be seen of this thing in this country. When the profession looks into these things, it will see that kindness will accomplish just as much.

Dr. Fillebrown: Everybody in this world will find some other body that will be influenced hypnotically by him. I know that Dr. McKellops can do anything that I can do. If there is any man on earth who hypnotizes his patients, it is Dr. McKellops. That is one of the surest ways of hypnotizing. Dr. Liebaud was asked once how he cured his little children? He said, "All I do is to take them in my arms, lay my hands on them, and they are cured."

Another excellent point he has made is, that practicing the art of hypnotism helps you. It forces you to kindness. No man can practice hypnotic influence or suggestion, unless he is kind and moderate, and careful with his patients, and that is one of the greatest lessons we learn. Dr. McKellops evidently did not take in the point of my paper. Hypnotism and McKellopsism are synonymous.

Dr. E. S. Chisholm, Tuscaloosa, Ala., said he was surprised that the subject should be so seriously discussed by intelligent men. He had not heard a single argument to demonstrate any science in it.

Drs. Poor, Pruyn, Roberts, Burnett, Taylor, Patterson and others, indorsed Dr. Fillebrown's position. Drs. Pruyn and Patterson suggested that such power should be exercised intelligently, and by specialists.

Dr. J. Rabe, Oakland, Cal., asked Dr. Fillebrown to give a practical demonstration. To this Dr. Fillebrown answered he was willing to do so, but that usually in such cases the operator did not like to take a new patient and one whom he had not seen before, but he would take some one as a dummy and show his mode of proceeding. Dr. Fillebrown then took one of the members and showed how he proceeded to practice hypnotism.

The Section then adjourned.

SECTION 2.—ETIOLOGY, PATHOLOGY AND BACTERIOLOGY.

Section called to order by the Chairman, Dr. G. V. Black.

A paper was read, entitled "Diseases of the Palate, with Reference to Dentistry: Their Pathology." (Illustrated with cases.) By Vida A. Latham, D.D.S., F.R.M.S., Chicago, Ill.

Diseases of the palate are not so rare as is often thought by physicians and dental surgeons, if attention be paid to that part of the mouth.

The anatomical position of the palate, by its structural continuity with the alveoli and gums in front, and its connection posteriorly with the tongue and pharynx by means of the pillars of the fauces, is liable to implication by the extension of disease from these parts; whilst, on the other hand, morbid processes originating in the palate may spread to the neighboring portions of the mouth, fauces, or pharynx.

Inflammation of the buccal tissue is not commonly found extending on to the mucous membrane covering the anterior edge of the coronoid process (the precoronoid space of Allen). Inflammatory action about the inferior third molar is readily transmitted to it, thence involving the soft palate. Conversely, ulcerations of the soft palate may tend to involve the tissues around the inferior third molar.

Other pathological conditions and complications were described; the diseases of the palate being grouped by the essayist as follows:

I. Congenital malformations, as cleft palate, which it is better to treat by itself as a subdivision of palatal disease.

II. Inflammation.

III. Ulcers.

IV. Necrosis, and

V. Tumors.

(Detailed description of numerous cases of palatal diseases which followed will be found in the paper as published in the Congress Transactions.)

In regard to the treatment, the author said: The chief danger in palatal operations is from hemorrhage—the ingress of blood into the trachea, especially when the patient is under ether or chloroform. Cocaine renders this danger much less, but gives another form of trouble by its uncertain action. The difficulties in differential diagnosis are many, and it requires much study to know the nature of the growths and to classify them properly, on account of their irregular morbid histology.

The second paper, entitled, "Some Facts—With Models—Showing the Relationship of the Dental Inter-Articulation with More or Less Obscure Pains about the Mouth and Jaws," by L. Van Orden, M.D., San Francisco, Cal., was read by the Secretary, Dr. Chisholm.

In this paper a number of cases of mal-occlusion, due to various causes, chiefly, injudicious extraction, resulting in various stages of pericementitis, were promptly relieved by the use of the corundum-stones correcting the occlusion, the conclusion being, in the words of the paper: "The main benefit that the writer has derived from the observation of these and other cases has been the conviction that the extraction of one or more teeth, especially of the inferior first molars, is liable, sooner or later, to lead to discomfort in some of the remaining teeth, and that such discomfort may, with patience, be located and relieved by the use of an engine-stone."

There being no discussion of the papers, the Section adjourned, *sine die.*

### SECTION 3.—CHEMISTRY AND METALLURGY.

This Section held no meeting.

### SECTION 4.—THERAPEUTICS AND MATERIA MEDICA.

Dr. N. S. Hoff, Ann Arbor, Vice-Chairman, in the chair.

The first order was the discussion of the paper by Dr. Poinsot, of Paris, France, on "The Extraction of the Pulps from Teeth in a Calcified State, by Trephining," (see page 595, Vol. XLVII.)

Dr. Cravens thought that the paper had suffered in translation, and on his motion, it was passed without discussion.

The next paper, "Experiments with Bichloride of Mercury," by Carrie M. Stewart, D.D.S., Ann Arbor, Mich., was read by the Chairman.

Because of the peculiar action of bichloride of mercury upon material of an albuminous nature, its efficacy as a germicide is believed by some scientists to be less than the standard usually assigned to it. When brought in contact with the substance to be acted upon, the albumen present is superficially coagulated, the interior of the mass escaping many times, because of a lack of penetrating power in the disinfectant. Such being the case, it has been urged that in the action of bichloride of mercury upon the germs of disease, the same effect is produced—*i. e.*—the external gelatinous capsule of the germ is coagulated, while the internal, vital portion is left untouched, and if by any means the compound between this covering and the disinfectant be dissolved, the micro-organism is quite as ready to manifest its characteristic phenomena as though it had never been subjected to the action of the disinfectant.

The paper contained a detailed history of a series of experiments in bouillon culture, using germs of the *staphylococcus pyogenes aureus and albus*, which although not spore-forming in the common acceptance of the term, are nevertheless very resistant to destructive influences.

"This work," the writer said, "was done to satisfy myself of the efficacy of bichloride of mercury as a germicide under certain conditions, and although the experiments made were comparatively few in number, they were accomplished with all possible care and accuracy."

The conclusion reached being that although bichloride of mercury is not sufficiently penetrating in its action to serve as a germicide, excepting under conditions most favorable to its complete performance of the results desired, yet the ideal germicide has not been discovered, and those that are so efficient as bichloride of mercury are hard to find. It would seem from the experiments of others, that the more concentrated solutions of this disinfectant are still less to be relied upon in their action than the strengths more ordinarily used, from the fact that the combination between the bichloride of mercury and the capsule of the germ is more quickly effected, and, although the portion influenced is probably of a firmer consistency than that brought about by using a solution of weaker strength, still the amount of penetration is not so great and the desired results not so nearly accomplished. Because of its extremely poisonous nature, bichloride of mercury will never be extensively used in dentistry, yet, with a proper knowledge of its powers and those of other germicidal agents of a high degree of usefulness, a combination may be effected in the future, which may prove of the utmost value to the oral surgeon.

The paper was, on motion, passed without discussion.

Then followed the reading of a paper, entitled, "Treatment of Abscess of the Maxillary Sinus," by Dr. E. Lecaudey, of Paris, France, which was read by Professor Barrie, of Paris.

He said: During the course of thirty years I have cured among my clients over sixty cases of abscess of the maxillary sinus. Having been a long time studying the subject, I once read a thesis by Dr. Veillard, treating of anal fistula, in which was described treatment with zinc chloride. The idea then struck me to apply the treatment to fistula of the sinus, and I obtained most excellent results.

The cases that I have oftenest treated were caused by the second bicuspid; less frequently, by the cuspid and first molar. The treatment has generally lasted from eight to twenty-one days, and has never lasted more than six weeks.

After having performed the extraction, I wash the cavity of the sinus with hydrogen peroxide; then I inject the following:

| | | |
|---|---|---|
| Chloride of zinc, | - - - | 1 gram. |
| Phenic acid, | - - - - | 5 " |
| Distilled water, | - - | 100 " |

In order to keep the fistula open, I place a little silk string, saturated with wax; and if, as rarely occurs, it will not close up, I take a small pencil of gutta-percha, which I saturate with a little chloride of zinc, place it for twenty-four hours inside of the fistula, and the edges close up by deep cicatrization very rapidly.

I have never had, owing to this method of treatment, any repetition of the disease.

Professor Barrie next read a paper by Mr. Denis, of Paris, France, entitled, "Boracine (Tetraborate of Soda)."

In one of the last numbers of *L'Odontologie* there appeared a most interesting article, treating of the antiseptic properties of boroborax.

For some time past I have used an identical process, and a similar substance called boracine. It is a salt, perfectly purified, resulting from the combination of equal parts of borax and boric acid. This tetraborate of soda is neither caustic, toxic, nor irritating; properties that often belong to antiseptics. Moreover, it has the advantage of being tasteless and odorless, and of dissolving in the proportion of sixteen per cent. I have tested the value of these products, and these are the observations which I have made. First, I have employed it for the disinfection of the dentinal tubuli, and I must say that the results were very satisfactory. I have also applied it in the treatment of the mucous membrane, in which case it has given me astonishing results.

The details of the treatment of several cases were given, the conclusion reached being: "I think, therefore, it would be a good thing to generalize the employment, particularly for the buccal mucous membrane, which interests us the most. In order to sustain such results, I could not do better than quote the names of Drs. Galezowski, Landolt, Hubert, LaGrange, etc., all of whom now employ boracine in all their clinics with great satisfaction."

There being a very small attendance at this Section, on motion the discussion of the papers was passed, and the Section then adjourned *sine die.*

### SECTION 5.—DENTAL AND ORAL SURGERY.

The Secretary-General, Dr. A. W. Harlan, was introduced to this Section by the Chairman, Dr. Brophy.

Dr. Harlan said, Mr. Chairman and Gentlemen: It has been impossible, on account of the numerous duties I have had to perform, to visit personally the various Sections, but to-day it has come my way to visit this Section, and from the reports that have been published from day to day we learn that some of the most valuable papers of the whole Congress have been presented here. And the object of my visit at this time is in the interests of surgery. Dr. Harlan then announced that Dr. Younger would give a clinic upon implanting one or more teeth, at the clinic rooms.

The Section then proceeded to the discussion of the topic: "What Neoplasms, both as to Kind and Degree, Necessitate the Excision of the Inferior Maxilla in whole or part when Associated with that Bone?"

Dr. Cryer, Philadelphia, Pa., opened the discussion by saying: I will classify diseases of the jaw into two kinds. Those of the inferior maxilla are either explainable or unexplainable. We know if we have the ordinary fistula situated below any of the teeth, we have to call it a dental alveolar fistula. If we have a tooth that is devitalized and an abscess below it, that is what I would class as an explainable disease. Then we can have growths coming up between the teeth which on the first appearance would appear to be non-explainable, but if by passing along the side we find this to be a fungoid growth that comes out of the tooth, it is certainly explainable. Another fungoid growth comes between the teeth where the teeth are healthy and are normal, and you can rest assured that this is not explainable. Of course, the unexplainable disease cannot always be cured, but if you know the cause it can be treated and cured. If you have one of those growths that is not explainable, do one of two things—cut it out, or let it alone. Don't use caustics. If you do it will break down the tissue and make it worse than before. Now, how far shall we remove the bone that is the seat of neoplasm? In any case remove the neoplasm. If it includes a small portion of the bone, remove it; if a large portion, remove a large portion; if it includes the whole jaw, remove it. I have a specimen with me which was pronounced a neoplasm. It was immediately behind a bicuspid. It puffed up and filled a greater portion of the mouth on one side until the patient could not close the mouth. The operation of a great majority of surgeons would have been, and it was recommended in this case, to remove half of the jaw.

Fortunately he fell into the hands of a man who came from the dental profession originally. He said: "No, remove that tumor and nothing more. Take off the main portion so you can see what you are doing, and then get down as far as it exists." The surgeon in this case cut away the large mass in the mouth and opened the lip at the median line and exposed the bone; he had his instruments ready to remove all if necessary, but he said: "We will go down first and cut out what we can with a circular saw, and then we will take the burs and smooth the part up, leave all the good tissue that is there and remove the bad." That section which I show you was removed, and we found we had not got all the diseased part out; burs were then used, and all the diseased bone, and a little more, was taken away, leaving a narrow rim of healthy bone with its overhanging edges of periosteum. When the bone is healthy, by all means leave it there, as was done in this case. This operation was performed eight years ago, and the patient has been acting on the stage all over the United States since that time.

Therefore, I wish to say again, cut away a neoplasm, and I might say a little beyond it, but no farther. Don't take out half a man's jaw if you can save even only a little rim of it. All who have seen this kind of operation, know that the bone to a very large extent will be reproduced, and then an artificial appliance can be placed in the mouth, and the man will be almost in a normal condition.

Dr. Younger, California, said: I have had to treat a number of cases, and found by the use of lactic acid I could remove the diseased bone without the use of the knife. I had a case where all the surgeons were against me. They were going to remove the jaw for a cancerous structure. The doctors said the only way was to remove the bone. When the lady came to me I found the pus discharging from different places, and I had been experimenting with lactic acid in similar cases, and so I suggested its use. I used lactic acid with about equal parts of warm water, as I find that is more effective than cold water. Afterwards I used a stronger solution. The operation was successful.

Dr. Brophy: Upon the closing of our Section, and the closing of the work which has been put before us to do, I feel that we have occasion to congratulate ourselves. A month or two ago it looked as though this Section would not be able to accomplish much. This afternoon I feel that when the records are prepared and published, the work of this Section will compare quite favorably with that performed in any other Section of the Congress. I feel that the work done here will reflect credit upon those who brought about this great meeting, and that our transactions when put in book form will fill an important place in the history of dentistry.

On motion of the Secretary, the Section adjourned *sine die.*

### SECTION 6.—OPERATIVE DENTISTRY.

The meeting was called to order by the Chairman, Dr. Jarvie.

A paper was read by Dr. Geo. W. Whitefield, of Evanston, Ill., entitled, "Conservative Method of Treating Fractures of the Anterior Teeth."

He said: As long as children play and stone sidewalks are hard, so long will teeth occasionally be broken off. Sometimes a whole tooth is knocked out or broken off at the gum line, but more often simply a corner is chipped. Again, the whole incising edge is snapped off far enough down to involve the pulp. What should be done in such cases? These accidents most often occur in childhood, so that we, as conscientious practitioners, must consider what is best, not only to remedy the present disfigurement, but also to secure the best results for a lifetime. For these young patients the extirpation of the pulp and placing a crown should should be the last resort.

Several cases in practice were then related. The Conservative Treatment of a Pulp exposed through the Fracture of a Central Incisor is thus given:

With bibulous paper I removed the blood from the exposed portion of the pulp, bathing it with a mild antiseptic, and after thoroughly drying the fractured end of the tooth, including the exposed portion of the pulp, I flowed collodion over the pulp, allowing it to extend a short distance beyond the exposure. After this was dry I repeated the operation two or three times, then before the last coat was quite dry, I covered the spot with a small piece of No. 4 gold foil. I repeated this operation with the collodion and gold foil several times; then being careful to remove all collodion from the margins, I flowed Justi's cement mixed to a cream over the end of the tooth, partially restoring its contour. Little more than two months later, finding the pulp alive and not very sensitive, I fitted an open-faced gold crown to the tooth.

In another case where a central incisor was so broken that when ground to a straight edge it was left one-eighth of an inch shorter than the other central, he proceeded as follows: I fitted a gold cap to the gingival margin, soldering a hook on the labial surface close up to the gum line.

At another operation I adjusted bands around the lateral and remaining central near the excising edge, each having a loop of hollow wire soldered on the labial surface. Taking a piece of fine piano wire I filed it down so that it would bend as a bow. Resting it in the loops in the bands on either side of the broken tooth, I sprung the piece of steel into the hook on the gold crown—a very weak spring was all that was needed to bring the tooth to the line with its mate. The moving of the tooth was accomplished in less than a week, with no disturbance to the pulp. This method is applicable to partially-erupted teeth; only in such cases it will be harder to adjust the crown.

Dr. Jarvie: At the meeting on Wednesday, there was a paper read on Oxyphosphates, and it was plain that to retard the setting of the oxyphosphate it was wise to use a bottle filled with ice-water. That was questioned by others in the room, among them by Dr. Knapp, of New Orleans, and he is prepared now to demonstrate the fact that it is impossible to properly mix the oxyphosphate on the outside of a bottle filled with ice-water.

Dr. Knapp said, that the moisture which condenses upon the outside of the bottle precludes the possibility of mixing cement on its surface. He said: The point I wish to make is, that no solution has yet been given by any of the members. It would be highly edifying to all of us to know how the crystallization of cement can be produced more slowly for setting bridge-pieces, particularly where there are three or four caps to be filled with the cement.

Dr. D. M. Cattell, of Chicago, Ill., read the following paper on "Operative Technics:"

The name "Operative Technics" is understood as the title of a department recently added to the curriculum of a few dental schools.

Since students generally began matriculating in dental schools, it has been noticed that students in the clinical department were not so well trained in the use of instruments at the beginning of their career at the chair as was desired, nor were those so apt who came first to college as those who had taken advantage of preliminary work under a preceptor.

So marked was this lack of manual dexterity, that when Dr. G. V. Black took charge of the infirmary of the Chicago College of Dental Surgery he immediately set about devising means for overcoming this deficiency, resulting in a paper before the Odontological Society of Chicago, June 21, 1888, entitled "Outlines of a Course of Study in Operative Dental Technics," and at the opening of the next school term in the institution with which Dr. Black was connected, the World's Pioneer Class in Operative Technics was organized.

Considering the newness of the course established, the inexperience of the instructor in that kind of work, the lack of practical experience as a guide to better effort, with no text-book applicable to any one of the many lessons gone over, the result was, in the highest degree, satisfactory.

The second year's work, with the experience acquired, was much easier, although many low places in the path had to be built up.

The third year was one of decided progress, owing to the fact that Prof. G. V. Black had issued his "Anatomy of the Human Teeth," which was just such a work as we had needed.

Five years have passed since the inauguration of the first class in dental operative technics. In that time much improvement has been made in the methods of teaching and systematizing the work.

In its beginning the course occupied three months' time; it is now lengthened to six months.

The technic department has for its aim four cardinal points:

1st. Manual training, or handicraft.

2d. System.—Each step following the other in methodical order.

3d. A greater familiarity with teeth.—Outward forms, inner channels, structure and plan of development.

4th. Individual reasoning.—Teaching students how to think for themselves.

The work of the department is divided into studies or lessons. Each division has its own heading, and notes of all work done under that heading must have its particular allotment of space in the note-book of each student. One study may be extended over a period of from three days to as many weeks, owing to the breadth and importance of the subject, or certain lessons may be continued and taken up again as opportunity presents. The present course, as given in the Northwestern University Dental School, is divided as follows:

1st. A study of technical terms, a hundred or more words with which the student will come in contact all through the lecture course and text-book reading. These words, when thoroughly understood, become the key by which students can often unlock and open to themselves the meaning of many otherwise obscure sentences. Examples: Abscess, ulcer, pulpitis, pericementitis, disinfectant, antiseptic, incise or incisor (scissors), septic, aseptic, caries, necrosis, mortification, gangrene, stimulant, anodyne, anesthetic, deciduous, mesial, distal, buccal, occlusal, etc.

2d. A study of typical tooth-forms, including their several surfaces and surface markings; noting certain "landmarks" peculiar to each denomination of teeth; also malformations. In this lesson each student has the work of Prof. Black, on the teeth, as a text-book. He is also supplied with a "ring of teeth"—a set of typical teeth properly arranged and strung on wire bent in the form of a ring for convenience in handling. When the study of a certain tooth is taken up the student has this tooth before him on the ring. There are also charts hung up in the class-room. These charts represent the same pictures enlarged as seen in the text-book, showing the several surfaces of each respective tooth. If possible, the student should have several teeth before him of the same denomination under discussion, that he may become familiar with the common variety of forms. After going carefully over the several surfaces and noting their lobes, developmental lines, sulci, and other face-markings, each student is then invited to select a tooth similar to the one undergoing inspection, from a miscellaneous lot of extracted teeth.

3d. A study of pulp chambers and canals. The student, having been supplied with a box containing some twenty or more wooden blocks one and one-fourth inches long by three-fourths inch thick, now fastens the tooth he has just selected, lengthwise on the block with sealing wax. The tooth and block can now be set in a small bench-vise. Two or more teeth of the same denomination are to be selected, and so arranged upon the blocks that different faces of the tooth will be presented to view. With selected files the student cuts away the tooth fastened on the block, so as to expose the pulp chamber and canal throughout its entire length; the cutting continuing until the central portion of the chamber is reached; he then removes the block and tooth from the vise. Now using the block as a handle he "inks" the exposed cut surface of the tooth, and presses it down upon a slip of selected paper, and the result is the so-called "silhouette pictures," showing the pulp chambers and canals. Of each tooth so dissected the student makes a line containing not less than five pictures. And of each denomination of teeth there should be several aspects of the chamber opened. When all the cuttings are made, the blocks to which they are attached are arranged in proper order in the original box, and kept by the student as a memorial of his early professional studies. The silhouette pictures of each denomination are printed on separate pages, of a specially-ruled blank book, and constitute when done a text-book, if you please, on the subject, that the student may keep for ready reference.

4th. The physical, anatomical, chemical and microscopical divisions of tooth structure. These studies are prepared by the means of lectures and charts, and when possible, having students make both longitudinal and cross sections, studying them through the microscope. This work for the freshmen should be rudimentary, as a more careful study of both hard and soft tissues will be had in the regular histological course. A special study of enamel in reference to cavity margins is made. The idea is to impress upon the student's mind the need of greater care in preparing enamel margins of cavities preparatory to filling.

5th. Free-hand drawing and modeling. The drawing of the different surfaces of the teeth by students helps to train the hand, as well as to call closer attention to form and certain surface markings. While the time is not sufficient for a thorough course in drawing, yet much may be done in the way of starting the student toward a better method of explaining or demonstrating a point in future before dental societies. Modeling teeth in clay, or its equivalent, is excellent training for both hand and eye, and should be indulged in as much as time will permit. The models should average at least six inches in length.

6th. A study of the more common medicaments such as are found generally in dental offices. This is not intended to supplant the systematic work of the chair of materia medica and therapeutics, but is preliminary to it, not going into detail other than to give the origin of the drug and the name of the dentist who proposed the remedy, if known, and his method of using it; also from whence the medicine is derived. Many of these remedies are compounds or mixtures. These remedies should be classified as disinfectants, antiseptics, anodynes or obtunders, stimulants, counter-irritants, etc. Each student should have in his case some eight or ten bottles, and these should be supplied with certain medicines for his experiments while in the class. These drugs are to be handled, looked at, smelled, tasted, indeed gotten thoroughly acquainted with.

7th. Students select from a miscellaneous lot of teeth placed before them a set, many of which should contain cavities ranging from simple exposure of dentine to large cavities involving pulp chambers. These teeth should be properly arranged as to original position, and their roots imbedded in gutta-percha. This set of teeth, so arranged, is to be considered a *dummy patient* for the student to practice upon. A wooden block carved to the shape of a human head, with a movable lower jaw, and instead of ridges to represent the alveolar processes there should be wide and deep grooves into which the gutta-percha that is to surround the roots of the teeth is placed. (Magnusson's Dummy Head is excellent.)

8th. Pulp-capping and devitalization. With the "dummy" before him, representing a supposed patient, the student seeks for slight pulp exposures; having listened to a lecture on the subject regarding the diagnosis, prognosis, treatment, and final capping or devitalization. As each pathological case is presented the student applies the remedies supplied in his case-bottles as directed by the instructor; after due treatment the supposed exposed pulp is capped or devitalized according to the specific directions of the instructor in charge.

9th. Supposed cases of dying pulps, putrefying pulps and entirely decomposed pulps. From the medicines furnished him each student applies treatments to the different conditions suggested, until he becomes familiar with the class of remedies required in each pathological condition.

10th. A study of the pathology and therapeutics of alveolar abscesses—blind or sleeping, acute, chronic, fistulous. Also of ulcers, pulpitis, pericimentitis, etc. This is done by lectures and charts; students making applications to supposed lesions from their medicaments.

11th. Cleansing, drying and filling root canals is practiced until students become quite familiar with the different methods of procedure; giving preference always to the one considered the best.

12th. Bleaching teeth. This study is made by the way of lectures and experiments in test-tubes or beakers upon vegetable matter, so that the results are soon observed by the students. Also practical application to discolored teeth found in the miscellaneous lot.

13th. Instruments. Each student supplies himself with a set of instruments and certain appliances according to a list furnished him. No other patterns are allowed. These instruments are a part of the set required of junior and senior students on entering the infirmary the following year. These instruments are classified and named. This classification and naming are according to certain rules recently proposed for the use of the school by Prof. G. V. Black. Students are furnished brass wire of a certain size, from which they are to make models of those called for by the list.

14th. Preparing cavities. Students are instructed regarding the opening up of cavities, and the proper shape they should assume. The simpler forms only are studied here—beveling of enamel-margins; what instruments should be used and how to grasp them. (No engine is allowed in this course.) *Hand-training* is one of the cardinal points. Ivory, bone, or certain compounds are often substituted for teeth in which to form cavities - typical shapes.

15th. Filling materials. Students practice filling these cavities with the different classes of filling materials—gutta-percha and the different preparations of the same; cements—both phosphates and chlorides; amalgams—alloys and copper, tin, aluminum, gold, both non-cohesive and cohesive, and combinations of two or more. These filling-materials are *studied*. Many of the cements and amalgams are made before the class.

16th. Miscellaneous matters. Any matter that may seem important that the freshman class should be posted in. These matters can be sandwiched in any time along through the course that the instructor deems best.

This course of technics may be somewhat modified each year, always with a view of bettering it. One essential condition of the course is, that nothing must be taught here that is not in accordance with the teachings of other special chairs; nothing taught that must be untaught by succeeding instructors; hence, the technic teacher should be familiar with the methods advocated by the other professors of the institution with which he is connected.

Now, if by means of this course of instruction students can be benefitted manually and mentally in a manner which is unattained by other known methods or systems of teaching, this effort will not have been in vain.

If the student has gained in handicraft, systematized his daily course of procedure, become more intimately acquainted with the organs on which his future labors will be bestowed, has learned the art of thinking for himself, and become logical in his reasoning, he is then ready to pass into the clinical department and there add practical knowledge to the hints he has thus far received.

DISCUSSION.

Dr. Carlton: I wish to make this suggestion in regard to cutting the sections: I precede the cutting by some instruction in instrument-making. I have the students make some delicate canal-instruments, such as canal-broaches, of piano wire. They are easily made, and if they spoil one they can easily make another. To any one who has ever cut sections, he will know it is quite easy to enter the pulp-chamber first; after having entered the pulp-chamber the broach can be inserted into the canal or canals, as the case may be, and this gives the student some training in the feeling of a broach in the canal. It gives them manual training, technical training in broaching canals; and the broach may be left in the canal and we may file until it is reached, which obviates the occurrence of that which I have found to be frequent with the novice—the destruction of the canal before they knew they had reached it. They strike the steel and they know they have reached the canal, and that warns them to be careful.

There is another point I think has not been sufficiently emphasized in the paper. That is instrument-making and sharpening. Those of us who have practiced in cities, and can get to the dental depot and get the most approved instrument at a moment's notice, do not appreciate how important it is to have such knowledge.

I think also the student should understand the quality of the material, so I have incorporated in my technics some lectures and instructions by demonstration of steel, what steel is, and the percentage of carbon that the instrument of steel should contain, the colors that indicate different degrees of hard temper, how to temper an instrument, and how to use a file in shaping an instrument. Then I give some little explanation by diagram of the different angles for the instruments in the cutting of different kinds of enamel or dentine, and for scraping.

Dr. H. A. Smith, Cincinnati: I want to bear testimony to the statement made in the paper, that the teaching of technics in the manner described is a real advance in the method of teaching, and I know the student that has taken this training comes to the chair of the patient a much more intelligent operator than without it. In the single matter of root-filling, about which we hear so much, the student who has mastered the cutting of teeth as laid down and taught now in colleges, will not meet the exaggerated difficulties which we hear.

Dr. G. V. Black said: This system is very young yet, and its full good has certainly not yet been demonstrated. The other department of prosthetic technics is going along with it, not in this discussion perhaps, but is following the same path and is doing the same good; good not only to the student of the school, but a great good to his patients who come to the school to be treated. For the sake of humanity, these boys require this training before they go to a patient.

Dr. Cattell, in closing the discussion, said: I have received many suggestions from Dr. Weeks, and many others who are interested in this work, and I want to take this occasion to thank all of those who have made such suggestions.

The Chairman: The Section of Operative Dentistry will now adjourn *sine die.*

(At the close of the meeting the representatives of eight schools interested in teaching dental technics met and organized the Association of Teachers of Operative and Prosthenic Technics. After electing Dr. D. M. Cattell, President; Dr. J. A. Dale, Secretary, and appointing a Committee on By-Laws, the meeting adjourned to meet at the call of the President.)

SECTION 7.—PROSTHESIS AND ORTHODONTIA.

The Section was called to order at 3 P. M., Dr. C. L. Goddard in the chair.

Dr. Geo. V. I. Brown, Duluth, Minn., read a paper entitled, "Prudence and Gutta-Percha, in Crown and Bridge-Work."

He said: My desire is to avoid, as far as possible, the detail of mechanical construction, and deal with the broad, underlying principles that govern the attachment of a foreign substance to a root in the living mouth, as a substitute for nature's perfect crown-work, and the yoking of one tooth to another to support substitutes, and calling upon them to perform duties belonging to the full number.

Experience has certainly given this class of work an unquestioned right to be considered among the useful and therefore reputable operations in the oral cavity; and the query naturally arises, "What has been the effect of this introduction upon the general practice of dentistry?" And a glance at the record seems to prove that the result has been a marked one both for good and for ill.

The benefits have been the preservation and restoration to a state of usefulness of many roots and broken down crowns of teeth that would otherwise have been lost, thus putting off the evil day when artificial dentures would be necessary.

Again, the result of this effort to utilize such roots has more than anything else influenced the wonderful advance in the therapeutic methods by which they are restored to healthful usefulness, and diseased conditions removed.

An advance has been made, in that while the average dentist was almost totally ignorant of the metal plate-work, to-day every student is required to have sufficient skill in this direction to be able at least to make an artistic-looking gold crown. On the other hand, instead of acquiring greater perfection in filling, the temptation has led to the wholesale destruction of natural crowns that might properly have been filled.

The principal points in crown-work are:

First: Attachment to secure without fear of dislodgment.

Second: A natural appearance if for the anterior teeth.

Third: Provision against the action of destructive agents at or near the gingival line.

Fourth: Secured against danger of splitting by a band, or its equivalent, by forming the joining surfaces of root and crown in such a manner as to equalize the strain.

Fifth: The imitation of the natural form of the crown upon proximal and occluding surfaces.

He said: I begin with the broad-sounding but quite true statement, that crowns inserted with any of the oxychloride or oxyphosphate cements, without band or other protection at the joining with the root, no matter how secure the pin may be, must be considered as of a temporary nature, for no matter how perfectly the surfaces may be adjusted, disintegration will surely follow, and not only cause loosening of the crown but allow more or less destructive action upon the root itself.

The use of amalgam as a setting is, of course, a great improvement upon cement in crowns of this class, but even amalgam is not always proof against bacterial influence at that critical point, the gingival line, as is shown by the frequent failure of fillings.

The teachings of Dr. Flagg and his followers, while perhaps going further than could be generally recommended, have at least been instrumental in establishing the value of gutta-percha in its capability to withstand the action of the acids of the mouth, and as a preservative against caries even where every other filling material would fail; its only disadvantage being that it wears away too readily in exposed positions; but while its good properties are of the first importance in crown-work, the softness does no harm, because it is protected from wear and will not disintegrate. The advantages may be summed up before proceeding to more detailed description under the following heads:

First. It is impervious to acid secretions or bacterial influences.

Second. Not irritating to surrounding tissues.

Third. Easily removed when necessary.

As against these three there is simply one consideration, viz: the difficulty of adjustment.

To one accustomed to use cement for this purpose, the difficulty of applying gutta-percha as a substitute seems almost insurmountable; but a moment's recollection of the trials attendant upon the setting of his first crowns will recall the fact that cement too had its difficulties for the beginner.

For gold crowns, where teeth are covered entirely, I prefer the red gutta-percha we get in the form of sheets. It has a degree of toughness that is valuable, but for anterior crowns with open faces the red line at the edge of the openings is undesirable, and I therefore use some white preparation that softens at a low temperature, as this property greatly facilitates its handling in the mouth. There being no danger to apprehend from wear, hardness is not so desirable as for filling.

It is my custom to heat both crowns and setting-material upon a tray to protect from the open flame, spread with warm instruments a thin coating over the inner surface of the crown, which should be first roughened on the inside by scratching with an instrument, aiming to have as nearly the exact amount necessary as possible, but depending upon a vent to get rid of a small amount of surplus; then after drying the surface of the teeth with alcohol and warm-air blast, wipe with some suitable germicide, saturate a pledget of cotton in eucalyptus, coat the surface with it to prevent the too rapid drying of the chloro-percha, of which a thick solution is then applied around the neck, so that when the crowns already heated as hot as they can be handled in the fingers, and prepared as described, are driven home, the eucalyptus, chloro-percha, and gutta-percha in the crowns will form a thick creamy mass that will penetrate and perfectly fill every space about the necks, and even if surplus be not removed afterward, will not cause irritation as might a particle of cement in the same position.

Sometimes too much gutta-percha will prevent the crown going quite into the proper position, just as sometimes happens with cement; but the remedy is much simpler, because easily removed while still warm; or if, for accidental or other causes, removal be desired afterward, hot water or hot air applied will in a short time heat the metal sufficiently to soften the material and facilitate the separation without the necessity of damaging the crown, and any one who has tried to pull them off without first warming them will be quite satisfied that any properly adjusted crown can be securely held in this manner.

After some discussion on this subject by Drs. Dunn, Matteson, E. P. Brown, C. L. Goddard, Oliver, Burne, of New South Wales, and W. P. Smith, Dr. Geo. J. Dennis, of Chicago, Ill., read a paper, entitled, "A Study of the Masticating Force of the Jaws."

He said: When he began his investigations of this subject he had hoped that some scientific knowledge might be gained of the stableness or unstableness of bridge-work, judged entirely from the mechanical standpoint. The subject was thoroughly investigated. Competent civil and mechanical engineers were consulted, but all acknowledged that the solution of the problem was entirely beyond them for these reasons:

1. Absolutely nothing was known concerning the resistance to strain, either direct or torsional, of the material (gold) used in forming the superstructure of the bridges. Its elastic qualities were also unknown quantities.

2. The supporting beam in dental bridges constantly varies in size and shape, so that no calculation could be made as with a square or triangular or even a rectangular beam of even size and shape throughout.

3. The varying density of the material of this superstructure, composed as it usually is of two, three, and four grades of gold, presented a combination upon which no calculations could be based.

4. The method of attachment of the superstructure to its abutments was an unknown factor and incapable of solution, for its strength must vary extensively with the peculiar conditions present in each individual case.

5. The strength of the superstructure was one of such great variability that nothing could be determined in regard to it.

After much investigation and research, he had constructed an instrument designed to contribute to a solution of the problem, by giving accurate information upon the amount of force exerted by the maxillary muscle in the closure of the jaws.

This instrument has a register of ninety-five pounds, and tests were conducted to determine the actual masticatory force of the maxillary muscles. So far but one person has been found who could accomplish the full register. From forty to sixty mouths have been examined. The range of the indicator, when placed between the molars of the adult jaws, was from sixty-five to eighty-five pounds, occasionally, but rarely, passing from eighty-five to ninety-five pounds. Between the bicuspids the force is found to diminish from five to fifteen pounds, the range extending from fifty to sixty-five pounds. Between the incisors the range extends from thirty to fifty-five pounds, showing a further diminution of about twenty pounds, as the pressure was applied at a greater distance from the location of the power.

Among children whose ages range from eight to fourteen years, a peculiar fact was noted in that the greater force was applied through the incisors rather than through the molars. The force exerted in these cases ranged from twelve to twenty-five pounds upon the anterior teeth, while upon the posterior the range was from ten to twenty pounds. The cause of this state of affairs is that during these years the permanent incisors have become firmly fixed in the jaws, while the temporary molars are being absorbed, their roots lacking the usual strength, which by reflection caused a less force to be exerted by the muscles of the jaw.

In the mouths of persons wearing plates, the amount of force was still less, ranging only from five to twenty pounds.

These figures apply to the greatest force that the jaws of persons of average strength exert. It is the belief of the writer, based upon experiments in his own mouth, that the actual pressure exerted by the jaws in the ordinary movements of chewing the food is only about one-half that of the figures given above, while in most cases in which the food is not properly masticated the force exerted will be found to be only one-third or even one-fourth that of the figures.

Thus it will be noted that our preconceived ideas in regard to the masticating force of the jaws have been greatly at variance with the actual facts. It is the hope of the essayist that the facts here set forth may be of service in the future in the study of strains placed upon fillings, crowns, and bridges, and all artificial dentures of whatsoever kind. As his observations become more extensive, further statistical information will be furnished through the medium of the dental journals.

There was no discussion, and the Section adjourned, *sine die.*

SECTION 8.—EDUCATION, LEGISLATION AND LITERATURE.

Dr. Patrick, the Chairman, called the meeting to order, and as there was not a quorum present, the motion was made and carried, to adjourn.

[*To be continued.*]

